# Supplementary material for: Effect of Peierls-like distortions on transport in amorphous phase change devices
Source: Commun Mater. 2025 Mar 29;6(1):56. doi: 10.1038/s43246-025-00776-5 (PMC11953050; doi:10.1038/s43246-025-00776-5)
Supplement: Supplementary file 2 — Supplemental Material [file 43246_2025_776_MOESM2_ESM.pdf]

Supplementary information for: Effect of Peierls-like distortions on transport in amorphous phase change devices

Nils Holle[1*], Sebastian Walfort[1], Riccardo Mazzarello[2], Martin Salinga[1*]

[1]University of Münster, Institute of Materials Physics, Wilhelm-Klemm-Str. 10, Münster, 48149, Germany.
[2]Sapienza Università di Roma, Department of Physics, Piazzale Aldo Moro 5, Roma, 00185, Italy.

*Corresponding author(s). E-mail(s): nils.holle@uni-muenster.de; martin.salinga@uni-muenster.de;

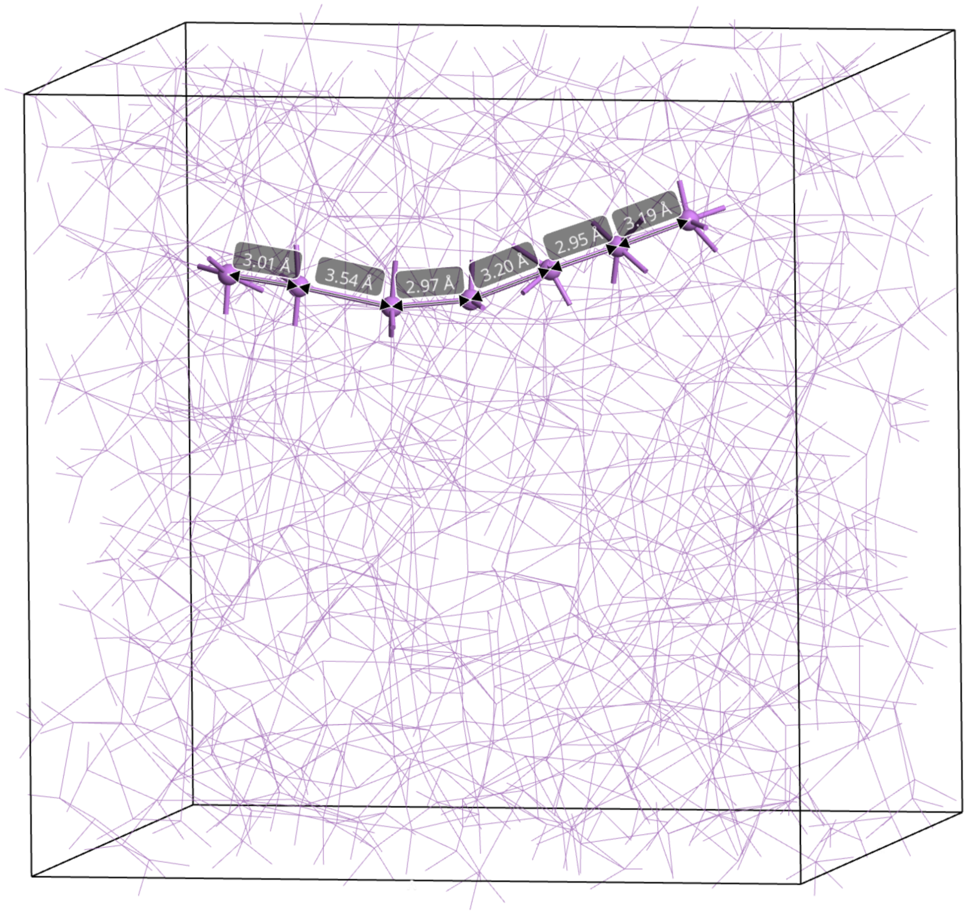

Supplementary Fig. 1: Exemplary configuration of amorphous antimony at 150 K, with a selected chain of atoms illustrating Peierls-like distortions in the amorphous phase. Throughout the structure, we find chains of alternating short and long bonds.

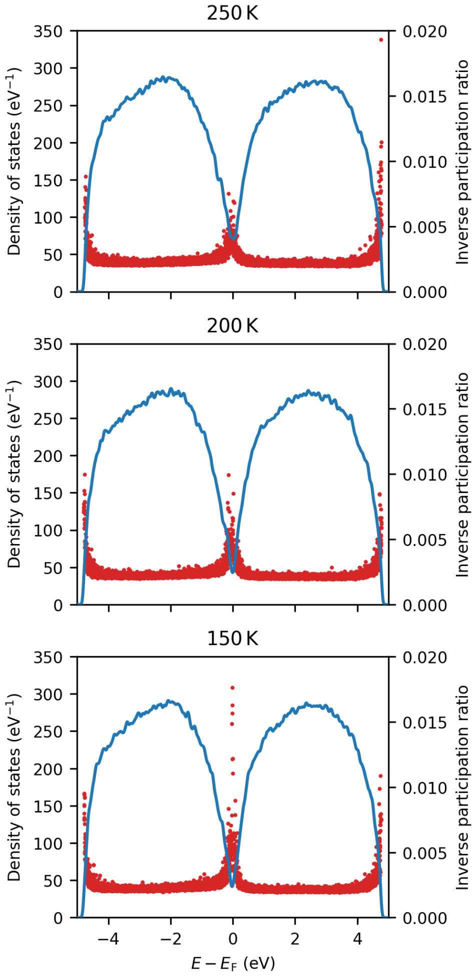

Supplementary Fig. 2: Calculations of electronic structure and localization for three different temperatures with a more accurate exchange-correlation functional show qualitatively the same results as the corresponding PBE-GGA calculations shown in the main text. At the lowest temperature of 150 K, we observe more states with exceptionally strong localization than for the PBE results (Fig. 3 a of the main text), which had to be expected, but not larger maximum values of the IPR. For the calculations shown here, we used the TB09 meta-GGA functional of Tran and Blaha [1], with $c \approx 1.13$ determined self-consistently. We employed an LDA PseudoDojo basis set and pseudopotential of medium quality [2], a $2 \times 2 \times 2$ grid of k-points, and a 150 Ha density mesh cutoff in all calculations.

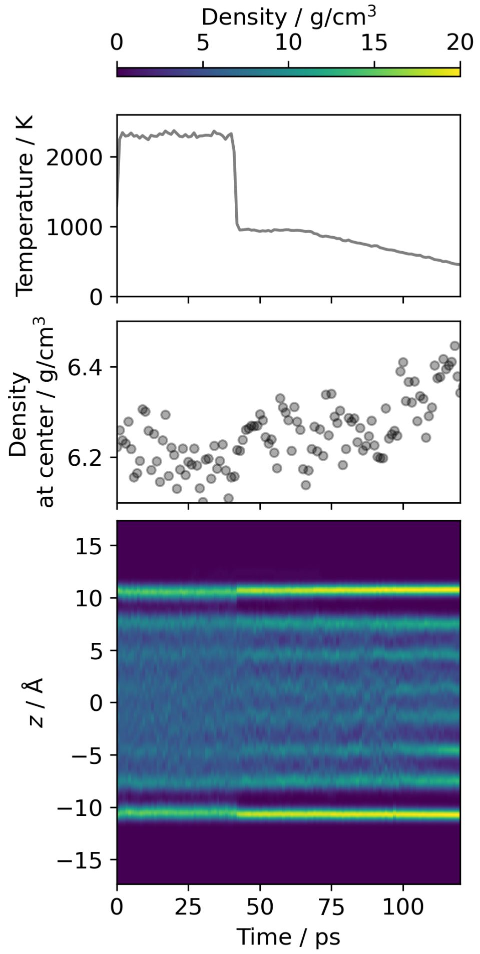

Supplementary Fig. 3: Formation of a layered structure in the amorphous thin film. Already at high temperatures above 2000 K, a dense wetting layer forms on top of the (100) tungsten surface that follows the BCC structure of tungsten (see Fig. 8). Spatial oscillations of density due to the confinement are also visible at high temperatures and become more evident at the melting point of antimony at approximately 900 K and below. The density at the center of the device ($z = 0$ in the lower sub-figure) increases at lower temperatures, which could be an indication of negative thermal expansion. This effect is also known for other phase change materials.

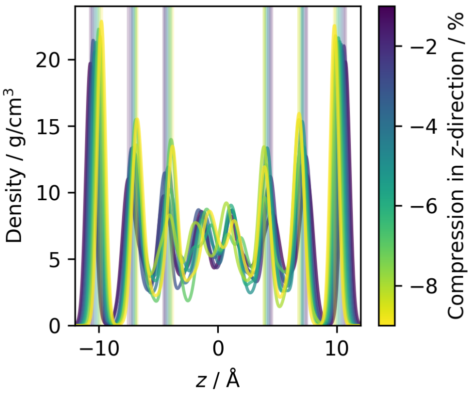

Supplementary Fig. 4: Peak positions in the mass density in Sb/W device structures in dependence of electrode distance. The electrode distance was reduced by the percentages encoded in colour, and additional melt-quench MD simulations have been performed. We observe both changes in the peak positions and changes in peak heights upon compression, but no signs of a disappearance of the oscillations in mass density if the system is given enough volume.

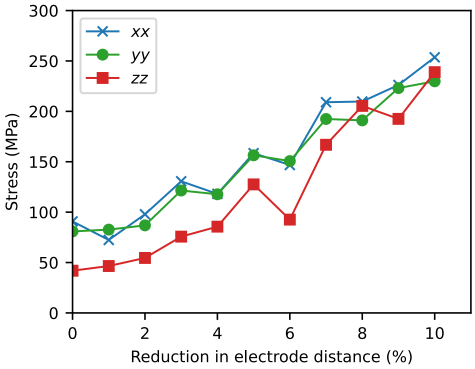

Supplementary Fig. 5: Diagonal elements of the stress tensor for our Sb/W device structures at 400 K in dependence of electrode distance.

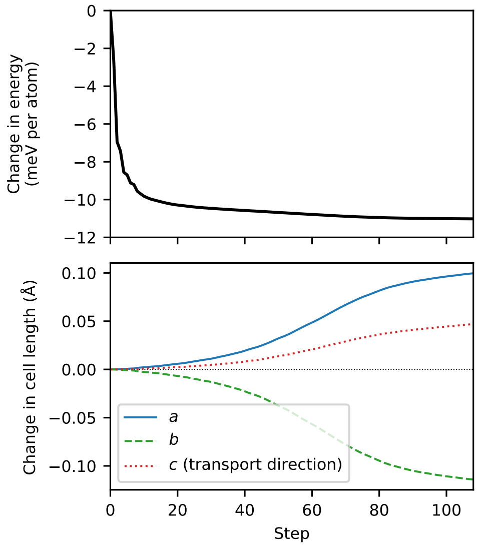

Supplementary Fig. 6: We find only small changes both in potential energy and in the cell parameters upon relaxation. The structure was relaxed using a BFGS optimizer until all forces were below a threshold of 50 meV/Å. To this end, DFT calculations were again performed using CP2k with the same settings as for the MD simulations shown in our manuscript. Instead of the more efficient orbital transformation method, we used a standard diagonalization scheme and also included Fermi-Dirac smearing with a width of 300 K.

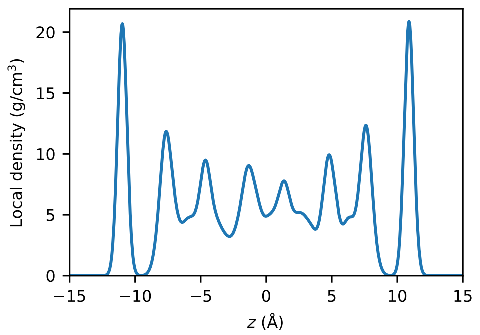

Supplementary Fig. 7: The oscillations in mass density persist after relaxation of both the simulation cell and atomic positions. Details of the structural relaxation are given in the caption of Fig. 6.

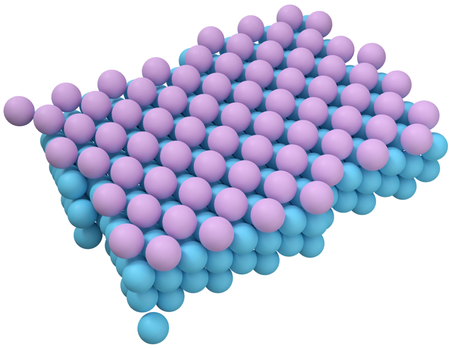

Supplementary Fig. 8: Antimony wetting layer on the tungsten (100) surface that forms already at very high temperatures (see Fig. 3). The layer follows exactly the BCC structure of tungsten, which is in line with the experimental results from [3].

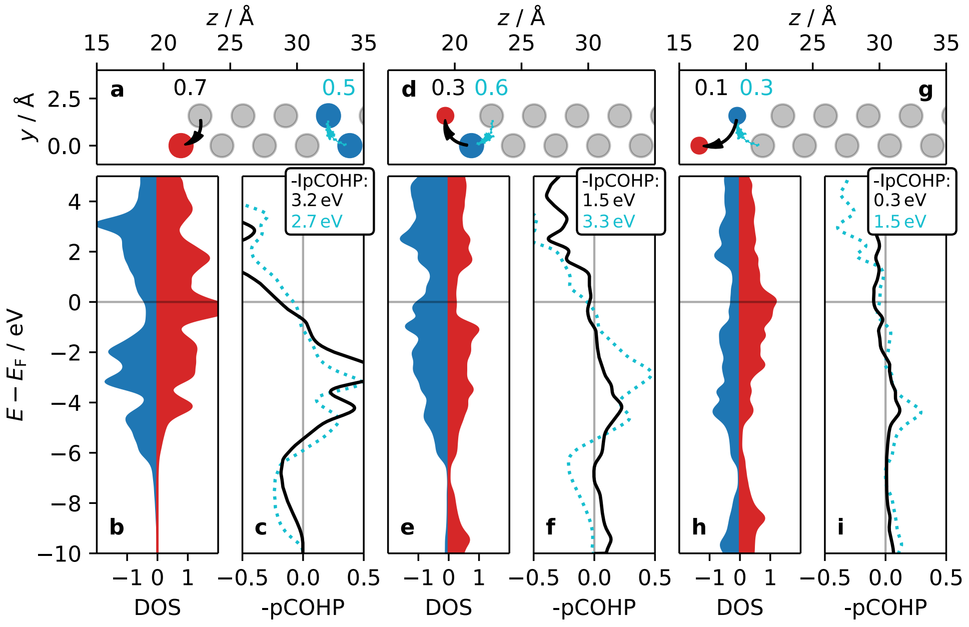

Supplementary Fig. 9: Bonding at the interface between a tungsten (100) surface and the first layers of antimony. While the first antimony layer is strongly bound to the surface, bonding to the second layer is much weaker. The left part of the figure shows data for the pristine tungsten surface. The calculations were performed using Quantum Espresso [4] and the Lobster code [5]. We used a 21 × 21 × 1 k-point grid and 50 Ry and 475 Ry cutoffs for the wave function and electron density, respectively. KJPAW pseudo-potentials (with 4f electrons of tungsten treated as valence electrons) were emploayed. For tungsten, the LCAO basis consisted of 5d, 5p, 6p, 5s, 6s, and 4f electrons. For antimony, 5p and 5s electrons were included. Atomic positions, where numbers give the integrated COBI value of a particular bond. The outermost tungsten layer is slightly "pulled" into the surface. (b) Projected DOS for the atoms marked in the corresponding colors in sub-figure (a). The outermost tungsten layer shows an increased number of states at the Fermi level. (c) Projected COHP curves and integrated COHP value for the two bonds indicated in sub-figure (a). The outermost bond is slightly stronger than a typical bond in bulk tungsten, but also shows larger antibonding contributions at the Fermi level. (d) Atomic positions and COBI values with one layer of antimony. The bonding between the two outermost tungsten layers does not seem to change drastically. The COBI value indicates that bonding to the first antimony layer has both ionic and covalent contributions. (e) Projected DOS for the atoms with corresponding colours in (d). (f) Projected COHP curves and integrated COHP value for the two bonds indicated in sub-figure (d). Bonding to the first tunsten layer is much weaker than the bond between tungsten atoms. The outermost W-W bond is strengthened by the presence of the antimony layer. (g) Same as (d), but with two layers of antimony. The bonding to the second layer of antimony is more ionic. (h) Same as (e), but for two layers of antimony. (i) Same as (f), but for two layers of antimony. The bond to the second layer of antimony is very weak and shows stronger antibonding contributions below the Fermi level. Bonding between antimony and tungsten is not affected by the presence of the second antimony layer.

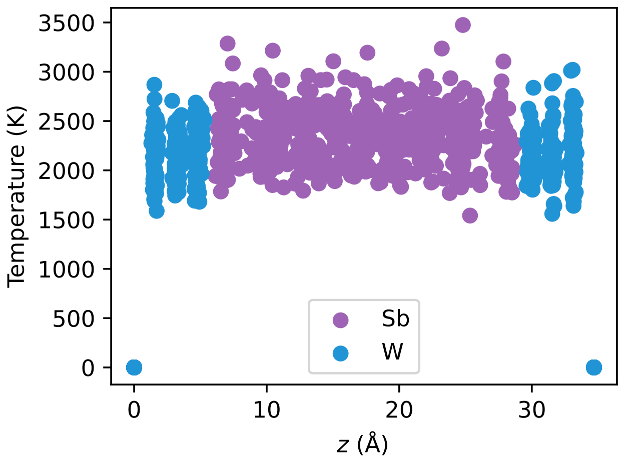

Supplementary Fig. 10: The temperature is kept constant across the whole device structure despite fixing the outermost layers. The data shows the temperature of each individual atom at a high thermostat temperature of approximately 2400 K. We do not observe a temperature drop in the outer tungsten layers.

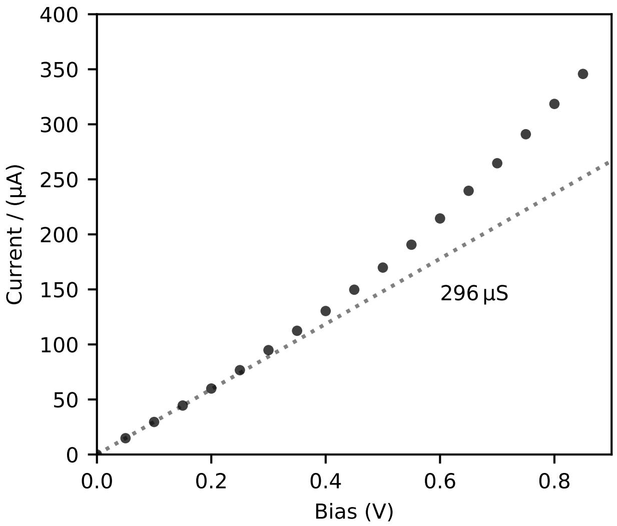

Supplementary Fig. 11: IV characteristics of the device structure suggests a conductivity of approximately 300 μS. At higher bias voltages, we observe a non-linear increase of the current, in agreement with experiments on other Sb-rich phase change materials [6].

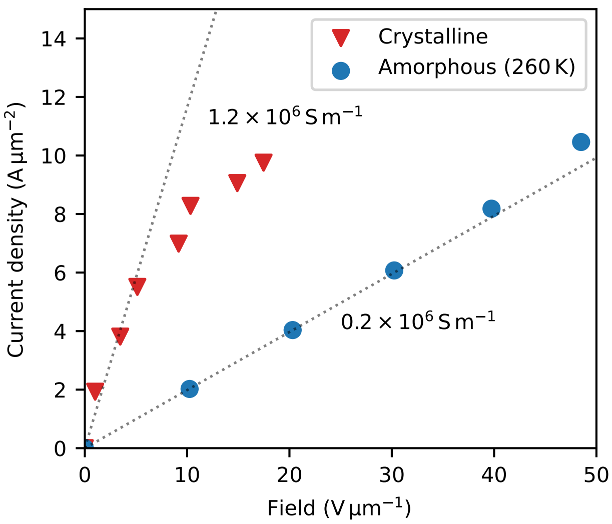

Supplementary Fig. 12: Comparison of the IV characteristics for crystalline and amorphous antimony shows a factor of six contrast in electrical conductivity. For this comparison, we simulated an additional device structure with crystalline antimony that is depicted in Fig. 13. Details of the calculation are given in the caption of that figure. We observe a conductivity of $1.2 \times 10^6\,\mathrm{S\,m^{-1}}$, compared to $2.5 \times 10^6\,\mathrm{S\,m^{-1}}$ in experiments. For the calculation of the electric field, we calculated the electrostatic difference potential at the respective bias, and its difference compared to the electrostatic difference potential at zero bias. We excluded the voltage drop over the wetting layer and the adjacent gap. The same calculation was performed for our amorphous device structure. Here, we observe a lower conductivity of $0.2 \times 10^6\,\mathrm{S\,m^{-1}}$. Our result corresponds to a specific resistance of approximately $7 \times 10^{-5}\,\Omega\,\mathrm{m}^2$. The contrast between crystalline and amorphous phase is much lower than in experiments, where a contrast of approximately two orders of magnitude was observed at 225 K. This point is discussed further in the main text.

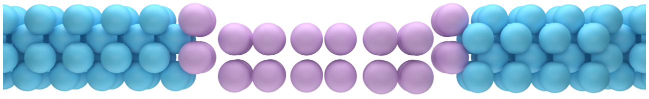

Supplementary Fig. 13: Visualization of the device structure simulated to obtain the data for crystalline antimony in Fig. 12. We study crystalline antimony on a tungsten (100) surface. The crystalline structure of antimony is inferred from partially recrystallizing the amorphous device structure in an additional constant temperature molecular dynamics simulation at 500 K, following melt-quenching to this temperature. We find a stack of layers of the pseudo-cubic (100) plane of antimony ($(01\bar{1}2)$ plane of the hexagonal lattice) on the tungsten surface. This is in full agreement with the experimental results from [3]. The formation of the wetting layer and bonding at the interface is studied in detail in Fig. 9. We created a device structure based on a $2 \times 2 \times 1$ repetition of the tungsten/antimony lattices in the lateral directions. Note the effect of Peierls-like distortions in the crystalline phase, which leads to an alternation of long and short bonds. This structure was then relaxed with a regular LCAO-DFT calculation in QuantumATK [7]. To this end, we used again the PBE exchange-correlation functional, a PseudoDojo basis set [2] of medium quality, a $7 \times 7 \times 1$ grid of k-points, and a 150 Ha wave function cutoff. We then used the relaxed device structure for additional NEGF calculations. In these calculations, we used the same FHI pseudopotentials (DZP for tungsten, DZDP for antimony) that we already used for the calculations of the amorphous tunsten/antimony systems. A $7 \times 7 \times 298$ grid of k-points was employed, where the latter number is only relevant for DFT calculations of the electrode.

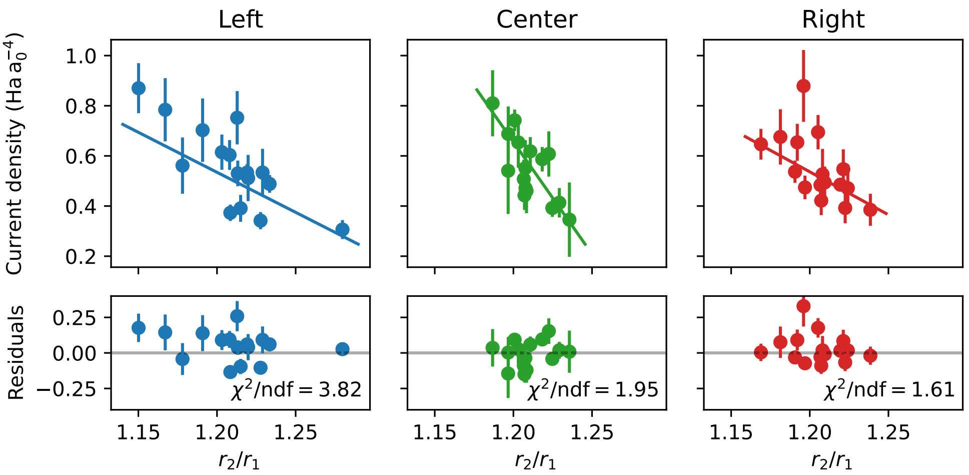

Supplementary Fig. 14: The influence of Peierls-like distortions on local current density can also be observed closer to the interface, but possibly with a less steep dependence. The figure shows the dependence of local current density on the local extent of Peierls-like distortions for the central layer of the amorphous thin film (Fig. 4 d of the main text), and the two outer layers next to the wetting layers. A decrease of local current density with increasing Peierls-like distortions is observed, albeit with a lower slope closer to the interface. This could be because the local current depends more strongly on few local contacts to the wetting layer in the outer regions of the amorphous structure.

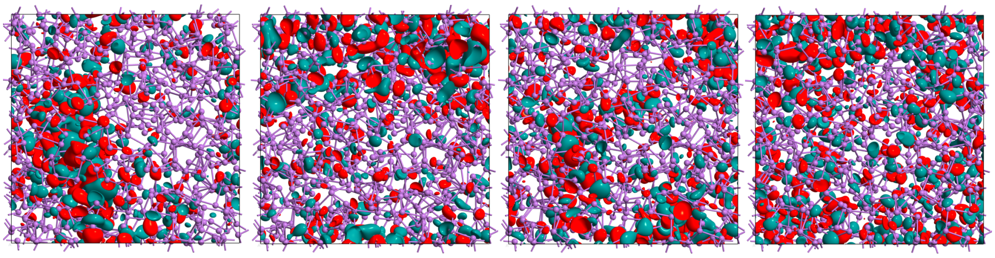

Supplementary Fig. 15: Isosurface plots of the four exemplary states shown in Fig. 3 of the main text, again in decreasing order of the IPR (left: most localized, right: least localized). The isovalue was set to $0.014\,\text{Å}^{-3/2}$ for all four states. Red and green colors indicate a phase of 0 and π, respectively.

Supplementary References

[1] Tran, F., Blaha, P.: Accurate Band Gaps of Semiconductors and Insulators with a Semilocal Exchange-Correlation Potential. Physical Review Letters **102**(22), 226401 (2009) https://doi.org/10.1103/PhysRevLett.102.226401

[2] van Setten, M.J., Giantomassi, M., Bousquet, E., Verstraete, M.J., Hamann, D.R., Gonze, X., Rignanese, G.-M.: The PseudoDojo: Training and grading a 85 element optimized norm-conserving pseudopotential table. Computer Physics Communications **226**, 39–54 (2018) https://doi.org/10.1016/j.cpc.2018.01.012

[3] Hopkins, B.J., Watts, G.D.: A RHEED study of the growth of antimony on clean tungsten (100). Surface Science **45**(1), 77–90 (1974) https://doi.org/10.1016/0039-6028(74)90156-3

[4] Giannozzi, P., Baroni, S., Bonini, N., Calandra, M., Car, R., Cavazzoni, C., Ceresoli, D., Chiarotti, G.L., Cococcioni, M., Dabo, I., Dal Corso, A., De Gironcoli, S., Fabris, S., Fratesi, G., Gebauer, R., Gerstmann, U., Gougoussis, C., Kokalj, A., Lazzeri, M., Martin-Samos, L., Marzari, N., Mauri, F., Mazzarello, R., Paolini, S., Pasquarello, A., Paulatto, L., Sbraccia, C., Scandolo, S., Sclauzero, G., Seitsonen, A.P., Smogunov, A., Umari, P., Wentzcovitch, R.M.: QUANTUM ESPRESSO: A modular and open-source software project for quantum simulations of materials. Journal of Physics: Condensed Matter **21**(39), 395502 (2009) https://doi.org/10.1088/0953-8984/21/39/395502

[5] Nelson, R., Ertural, C., George, J., Deringer, V.L., Hautier, G., Dronskowski, R.: LOBSTER: Local orbital projections, atomic charges, and chemical-bonding analysis from projector-augmented-wave-based density-functional theory. Journal of Computational Chemistry **41**(21), 1931–1940 (2020) https://doi.org/10.1002/jcc.26353

[6] Kaes, M., Le Gallo, M., Sebastian, A., Salinga, M., Krebs, D.: High-field electrical transport in amorphous phase-change materials. Journal of Applied Physics **118**(13), 135707 (2015) https://doi.org/10.1063/1.4932204

[7] Smidstrup, S., Markussen, T., Vancraeyveld, P., Wellendorff, J., Schneider, J., Gunst, T., Verstichel, B., Stradi, D., Khomyakov, P.A., Vej-Hansen, U.G., Lee, M.-E., Chill, S.T., Rasmussen, F., Penazzi, G., Corsetti, F., Ojanperä, A., Jensen, K., Palsgaard, M.L.N., Martinez, U., Blom, A., Brandbyge, M., Stokbro, K.: QuantumATK: An integrated platform of electronic and atomic-scale modelling tools. Journal of Physics: Condensed Matter **32**(1), 015901 (2020) https://doi.org/10.1088/1361-648X/ab4007
